# Knowledge, Attitudes, and Practices towards Cervical Cancer and Screening amongst Female Healthcare Professionals: A Cross-Sectional Study

**DOI:** 10.1155/2019/5423130

**Published:** 2019-10-17

**Authors:** Humariya Heena, Sajid Durrani, Isamme AlFayyad, Muhammad Riaz, Rabeena Tabasim, Gazi Parvez, Amani Abu-Shaheen

**Affiliations:** ^1^Research Center, King Fahad Medical City, Riyadh, Saudi Arabia; ^2^Comprehensive Cancer Center, King Fahad Medical City, Riyadh, Saudi Arabia; ^3^Department of Statistics, University of Malakand, Chakdara, Lower Dir, Pakistan; ^4^Women's Specialized Hospital, King Fahad Medical City, Riyadh, Saudi Arabia; ^5^Department of Anaesthesia, Sheikh Khalifa Medical City, Ajman, UAE

## Abstract

**Background:**

Cervical cancer is a potentially preventable disease if appropriate screening and prophylactic strategies are employed. However, lack of knowledge and awareness can result in underutilization of the preventive strategies. Healthcare professionals with adequate knowledge play a huge role in influencing the beliefs and practices of the general public in a positive way. We assessed the knowledge, attitudes, and practices of cervical cancer and screening amongst female healthcare professionals at King Fahad Medical City (KFMC), Saudi Arabia.

**Methods:**

We conducted a cross-sectional study on female healthcare professionals at KFMC. Data were collected using a predesigned, tested, and self-administered questionnaire. The questionnaire included specific sections to test the participants' knowledge, attitude, and practices related to cervical cancer and its screening. Data analysis was done using descriptive statistics.

**Results:**

Data from 395 participants were included in the final analysis. The majority of the study participants were nurses (*n* = 261, 66.1%). The mean age of the participants was 34.7 years and 239 (60.5%) participants were married. Only 16 (4.0%) participants appeared to have good level knowledge of cervical cancer (in terms of risk factors, vulnerability, signs and symptoms, ways of prevention, and ways of screening) and 58 (14.7%) participants had fair level knowledge. A total of 343 (86.8%) participants believed that Pap smear test is a useful test for the detection of cervical cancer and 103 (26.2%) participants had undergone Pap smear testing.

**Conclusions:**

Our study population showed poor knowledge of cervical cancer as a disease. The participants had a fair knowledge of Pap smear testing, but only a quarter of the cohort had undergone testing themselves. This study highlights the need for formal educational programs for the healthcare workers at KFMC specifically to improve their knowledge regarding the risk factors and early signs and symptoms of cervical cancer.

## 1. Background

Cervical cancer is the fourth most common cancer and also the fourth leading cause of cancer-related deaths in women globally [1]. In Saudi Arabia, cervical cancer is the eighth most common cancer amongst women in the 45- to 59-year age group and accounts to 2.2% of all cancers [2]. According to the fact sheet of human papillomavirus (HPV) information center (2018), it is estimated that, every year, 316 women are diagnosed with cervical cancer and 158 die as a result of the disease in Saudi Arabia [3]. Several common risk factors recognized to be associated with cervical cancer worldwide include sexually transmitted diseases (mainly HPV and others human immunodeficiency virus, herpes simplex virus), reproductive and sexual factors (multiple sexual partners, early age at the first sexual intercourse, early age at first delivery, parity, and oral contraceptive pills), behavioral factors (smoking and obesity), and host factors (genetic sensitivity) [4]. Abnormal vaginal bleeding, foul-smelling vaginal discharge, and contact bleeding are recognized as the major signs of cervical cancer, and in many cases, women with cervical cancer report no symptoms.

Almost all cervical cancers are caused by HPV and, therefore, are largely preventable [5]. Over the past several decades, the incidence of cervical cancer has decreased in developed countries [6, 7]. This is mainly attributed to increased awareness and more effective screening and prevention strategies employed in these countries [8, 9]. In addition, the HPV vaccine has contributed to a decline in the incidence rate of cervical cancer [10]. Three types of tests are currently available and are widely used for the screening of cervical cancer. These include tests for HPV, cytology-based Papanicolaou test (Pap test), and unaided visual inspection with acetic acid (VIA) [11]. However, public awareness of these tests especially in developing countries is limited [12]. HPV 16 and 18 are the most common subtypes of HPV causing cervical cancer and are responsible for most of the cervical cancers worldwide [5]. The association of cervical cancer and HPV infection implies that cervical cancer can be prevented by HPV vaccination. Consequently, HPV vaccines have been developed [13]. In Saudi Arabia, two vaccinations against HPV, bivalent vaccine (Cervarix) and quadrivalent vaccine (Gardasil) were approved in the year 2010 for females between the ages of 11 and 26 years.

While all these developments in the prevention and screening of cervical cancer are taking place, it is imperative that the benefits are utilized by all women including those living in the developing countries. Having good knowledge and awareness will help in ensuring that the disease burden does not increase. One concerning aspect is that most patients with cervical cancer in Saudi Arabia present at advanced stages leading to adverse outcomes [14]. Moreover, it has been found that the cost of treating late-stage cervical cancer is substantially higher than that of early-stage cancer [15]. Screening helps in the detection of cancer at an early stage when it can be treated more effectively. The late presentation of patients with cervical cancer in Saudi Arabia could be due to lack of knowledge and awareness leading to inadequate screening virtually nonexistent screening mechanisms for early detection [14]. Moreover, the decision to undergo screening highly depends on the healthcare professionals involved as well as the patient [16]. As per the results of World Health Survey Plus conducted in 2008/2009, only 7.6% of women in the 25- to 49-year age group in Saudi Arabia had a Pap smear test done, thus emphasizing the need to spread awareness amongst women [17].

Prognosis can be improved if screening is embraced and widely employed. For this, it is important that the healthcare workers are educated and well aware so that they can influence the beliefs and actions of the general public. Many studies have been conducted in other developing countries to gauge the knowledge and awareness about cervical cancer and to study the extent of utilization of the screening methods [12, 18–22]. In Saudi Arabia, although a number of studies have been conducted to assess the knowledge and practices of cervical cancer screening in the general population, especially in young girls, studies on healthcare staff have been few [23–25]. Healthcare facilities are available free of cost in Saudi Arabia and it is important that these facilities are utilized well, which can help in improving the general health status of the nation. Healthcare workers can play a central role in raising awareness of the general public, and therefore, their knowledge needs to be assessed and updated on a regular basis. In addition, in Middle Eastern countries, in particular patients seeking medical care prefer to have women as their caregivers with several studies providing traditional and religious beliefs as the main reason. Women are most likely to feel comfortable to talk about their symptoms with a female only. Even female healthcare providers are hesitant to talk about these issues with male physicians.

We, therefore, conducted this study to assess the knowledge, attitudes, and practices towards cervical cancer and screening amongst female healthcare professionals at King Fahad Medical City (KFMC), a tertiary hospital in Riyadh, Saudi Arabia.

## 2. Methods

### 2.1. Study Design and Data Collection

We conducted a cross-sectional study at KFMC in 2018. The study population included female healthcare workers with at least one year of clinical experience, including physicians, nurses, and allied health staff. The questionnaire was developed from previously published studies after an in-depth literature review [15–21] and then validated through experts. As per the guidelines, a committee of 2 experts each in research methodology, obstetrics and gynecology, and oncology further confirmed the validity of the questionnaire before the pilot study. Eight questions related to signs and symptoms were modified, and 5 were deleted as they were either not applicable to healthcare workers or not related to this topic as per the expert comments. After that, pilot testing was done on the prefinal version with 70 participants to assess the clarity of the questionnaire. Results of the pilot and the current study showed that Cronbach's alpha was >0.70.

The study population was stratified according to their professions into three groups: physicians, nurses, and allied healthcare workers.

To ensure appropriate and equal representation from each group of healthcare professionals, proportionate sampling method according to the profession was adopted to derive a sample with equal representation 4 : 1 : 1. 260 nurses (out of 2400), 65 physicians (out of 600), and 65 allied healthcare workers (out of 700) were selected and approached on a random basis from each hospital/center/administration at KFMC. The total sample size was determined to be 390.

The study participants were randomly selected from each profession. A survey cover sheet explaining the study was attached to the questionnaire and the ones who signed it went to the next step of questionnaire completion. Subject identifiers were not used in the questionnaire, and hence, confidentiality was maintained. A trained research assistant enrolled voluntarily willing participants who filled in the questionnaire and returned it.

The questionnaire was designed to include all information such as sociodemographic characteristics, knowledge, attitude, and practice of cervical cancer screening.

Participants' knowledge of cervical cancer was assessed by listing questions related to risk factors, number of sexual partners, early sexual intercourse, HPV infection, cigarette smoking, and other vulnerable factors in women. Questions under the five items asking about risk factors, vulnerability, signs and symptoms, prevention, and ways of screening for cervical cancer were also included.

For each item, the participants were asked to choose one of the three options: “Yes,” “No,” or “Don't know.”

The scale was then dichotomized such that “Yes” was considered as 1 and No/Don't know as 0. A total knowledge score for all the items was computed by adding up (maximum score of 20). The total score was then categorized as poor knowledge (score of 0–4), fair knowledge (score of 5–10), and good knowledge (10–20).

Participants' attitude was assessed by asking them to rate each of the following statements on a 5-point Likert scale: (1) carcinoma of the cervix is highly prevalent and is a leading cause of deaths amongst all malignancies in Saudi Arabia; (2) any young woman including you can acquire cervical carcinoma; (3) carcinoma of the cervix cannot be transmitted from one person to another; (4) screening helps in prevention of carcinoma of the cervix; (5) screening causes no harm to the client; (6) screening for cervical cancer is not expensive; and (7) if screening is free and causes no harm, will you screen?

Respondents were asked to choose one of the following options for each of the statements listed above: “strongly agree,” “agree,” “neither agree nor disagree,” “disagree,” or “strongly disagree.” For ease of presenting results, responses for “strongly agree” and “agree” and for “disagree” and “strongly disagree” were combined.

Participants' practices were assessed by asking specific questions about practices regarding cervical cancer screening. Respondents were asked whether they had heard of Pap smear test and whether they believe it is a useful tool for early detection of cervical cancer. They were further asked whether they had undergone Pap smear testing, at what interval they get it done, steps to be taken if any abnormality was found in Pap smear test, and reasons if they had not undergone Pap smear. The questionnaire also enquired about other details of Pap smear testing such as the best time for doing the test, who should do it (doctor/trained nurse), how it is done, and benefits of the test. Participants were asked whether they believe undergoing Pap smear testing is a good practice and also whether they believe it is a painful test. Finally, they were asked whether they were aware that HPV vaccination is done in their hospital and whether they had been vaccinated for HPV.

The study was approved by the ethical committee at the KFMC, and informed consent was obtained from each participant before enrolment.

### 2.2. Statistical Analysis

Demographic characteristics, knowledge, attitude, and practice of cervical cancer screening were described using descriptive statistics including percentages, frequencies, mean, median, standard deviation (SD), and interquartile range (IQR). All analyses were conducted using the software STATA version 12.

## 3. Results

### 3.1. Sociodemographic Characteristics

Of the 420 questionnaires that were distributed, 395 (94%) were returned and included in the analysis. The mean age (SD) of the participants was 34.7 (8.3) years. A total of 261 (66.1%) respondents were nurses, 63 (16.0%) respondents were physicians, and the remaining 71 (18.0%) respondents included pharmacists, dieticians, technicians, health educators, physiotherapists, and therapists. About 60% of the respondents were married. Nine (2.3%) participants reported having history of cervical cancer (Table 1).

### 3.2. Participants' Knowledge about Cervical Cancer

Many of the participants were not knowledgeable about cervical cancer. For example, only 8.9% of the sample knew that multiple sexual partners placed a woman at risk for cervical cancer. Women older than 50 years of age are at higher risk, yet only 8.6% of the sample had that knowledge. In advanced stages of cervical cancer, sign and symptoms a woman may experience are vaginal bleeding, foul-smelling vaginal discharge, and contact bleeding. However, a majority of the participants were lacking knowledge (93%, 92%, and 87%), respectively. As for preventing cervical cancer, 90% of the participants were unaware of the major behaviors one could do or avoid to prevent cervical cancer. Lastly, a majority of the participants did not have knowledge about the different ways of screening for cervical cancer (Table 2).

### 3.3. Participants' Attitudes towards Cervical Cancer

The majority of participants showed disagreement for all the statements in this section. More than three-fourths of the participants (84.8%) disagreed with the statement “screening helps in prevention of carcinoma of the cervix”. Participants' responses on the various statements that were designed to test their attitude towards cervical cancer are listed in Table 3. Overall, only 15 (3.8%) respondents agreed that they would have screening done if it was free and caused no harm.

### 3.4. Practice and Knowledge of Cervical Cancer Screening

Although 343 (86.8%) participants believed that Pap smear test is a useful test for detection of cervical cancer, only 103 (26.2%) participants had undergone Pap smear testing. Further, 18.7%, 43.8%, and 29.6% of the participants believed that Pap smear test should be started at the age of 20 years, 30 years, and after menopause, respectively. Sixty-three percent of the respondents agreed that the best time for a Pap smear test is a week after period, and 76.2% believed that Pap smear testing should be done by a doctor. Also, 78.9% of the respondents agreed that further tests should be done if any abnormality is detected in Pap smear test. Only 56 (14.2%) respondents were aware that HPV vaccination is available at KFMC and 22 (5.6%) had been vaccinated for HPV.  Table 4 presents the results of participants' responses to questions on knowledge and practice of cervical cancer screening.

Table 5 shows the relation of the total score for knowledge about cervical cancer (categorical) with demographic variables.

The total scale score was significantly negatively correlated with age (*p* value = 0.002) and the total number of years of experience (*p* value = 0.004). When we used logistic regression with binary variable (fair-good knowledge = 1 and poor = 0) as a dependent variable and categorical age (≤30 years vs. >30 years) as explanatory, younger age was significantly associated with the higher odds of having fair to good knowledge odds ratios (OR = 2.22, 95% CI (1.31–3.78).

However, when categorized, the years of experience (<10 years vs. ≥10 years) did not stand significant (Table 5).

## 4. Discussion

Progress in the understanding of cervical cancer has helped recognize its preventable nature [5]. It is well established that HPV vaccination and adequate screening can reduce the burden of the disease to a great extent [6, 7]. For effective screening and prophylaxis, it is of utmost importance to understand the knowledge, perceptions, and beliefs of the population especially that of the healthcare staff as they constitute an important source of propagation of health-related information. Many studies conducted in the developing countries have shed light on the level of understanding and knowledge of the population, which could provide useful information to the healthcare systems to develop appropriate educational strategies [19–21].

Our cohort scored very poorly on the knowledge score, which is quite alarming as basic level of knowledge about common diseases is expected from the healthcare staff. Adequate knowledge is an important determinant of positive attitude and because our study population did not have the knowledge, their attitude and beliefs were also concerning. On the other hand, participants' knowledge about the screening of cervical cancer was fairly good. Most respondents believed that Pap smear is a useful tool for early detection of cervical cancer. They also seemed to have a fair knowledge about certain aspects of Pap smear testing such as timing of doing Pap smear, action to be taken if the Pap test results are positive, and so on.

In a study conducted on medical students in Al-Ahsa, about fifty percent of the study participants were aware of the early signs and symptoms and risk factors of cervical cancer [26]. The better knowledge of these students compared to our cohort can be explained by the fact that these students, especially those in the final year, must have been taught about cervical cancer as a part of their curriculum recently and therefore were more informed about the disease. This implies is that medical students are not educated about cervical cancer in any depth. Therefore, they are most likely not informed prior to practice. It reflects that there are no continuing medical education activities regarding cervical cancer screening and prevention for healthcare professionals in the region. Moreover, there is a low incidence of cervical cancer in Saudi Arabia due to which the healthcare professionals come across few cases with cervical cancer, and therefore, they have inadequate knowledge regarding HPV vaccine and other prevention modes [27]. This highlights the fact that continued medical education is imperative to help the healthcare staff to keep abreast of the facts about important diseases as well as to keep them updated with the newer developments related to healthcare.

When formal screening programs are not in place, cervical screening is mostly “opportunistic,” which means women attending the clinics for other ailments are directed by the healthcare workers for cervical cancer screening. This is mostly done by staff in the gynecological department; however, if healthcare workers in other specialties also start referring eligible patients, the number of patients undergoing cervical cancer screening will greatly increase [23, 28].

Most women in our cohort were young and married making them most likely sexually active. Although multiple sexual partners are a risk factor for cervical cancer, we did not include that personal question to the participants, believing it to be offensive to the culture in Saudi Arabia. It is highly recommended to have Pap smear done in women above 21 years of age [29]. Also, HPV vaccine can be taken by women until the age of 26 years [30]. Gardasil (quadrivalent vaccine effective against HPV types 6, 11, 16, and 18) and Cervarix (bivalent vaccine effective against HPV types 16 and 18) are the two vaccines approved by the US FDA in 2006 and 2009, respectively [30, 31]. These are widely used in western countries, and their use should be encouraged in developing countries. These vaccines were approved in Saudi Arabia in the year 2010 for females between the ages of 11 and 26 years [13]. Vaccination for HPV could not be included in Saudi's national vaccination programs viewing the low incidence and higher cost for the health system. Thus, it is presently offered to selected individuals including women confirmed at risk for HPV infection or who voluntarily want to be vaccinated [32]. These vaccines are available in Saudi Arabia in select hospitals. KFMC is one such hospital and young female staff should take advantage of this facility and protect themselves from HPV infection. From the results of our study, it appears that most of our staff are not aware that this vaccine is available within KFMC. This calls for an urgent need to plan strategies to educate the staff not only on the benefits and need of HPV vaccination but also about its availability within the KFMC facility.

A study was conducted in Riyadh, Saudi Arabia, to examine whether the educational program had any effect on female healthcare students in terms of their knowledge regarding screening and prevention of cervical cancer. The results were promising with all scores improving significantly after the intervention in the form of educational program. This study provides further insight into the necessity and importance of educational activities, which could be in the form of lectures using audiovisual aids [24].

Our study has several limitations. Firstly, it was conducted only in one center, and therefore, the results cannot be generalized to the healthcare workers at other institutions in Saudi Arabia. Secondly, our sample was composed of a heterogeneous group of healthcare workers including physicians, nurses, and allied healthcare workers in various specialties which may have led to varied knowledge, attitude, and practices towards the screening. It is sad that healthcare workers especially nurses and physicians lacked the knowledge they should have had from their formal education.

The study results imply that if women, irrespective of their profession, do not receive education to increase their functional knowledge, understanding, and acceptance of routine cervical cancer screening, then they may not be able to promote behavior change in themselves, in their patients, or in the general population. First and foremost, the institution needs to develop a plan to intervene through education for self-care and also encourage them to educate other women they may encounter in their professional role or in their personal life. Also, a follow-up study can be considered to evaluate the usefulness of the educational program after it is in place at KFMC.

Further studies should be carried out in other hospitals in Saudi Arabia to understand the gaps in the knowledge and practice of cervical cancer screening.

## 5. Conclusion

Cervical cancer is totally preventable. Healthcare providers should be proactive in promoting women's health and preventing disease. Therefore, making sure healthcare professionals are informed about the benefits of routine cervical cancer screening and preventive methods will be a step toward making sure cervical cancer does not increase in Saudi Arabia.

## Figures and Tables

**Table 1:** Participants' sociodemographic characteristics.

Variables	Mean (SD)/median (IQR)^a^
Age^*∗*^	34.7 (8.3)
Experience in years^*∗*^	10 (6–16)
Age at marriage^*∗*^ (*n* = 252)	26.2 (4.4)
Number of pregnancies^*∗*^ (*n* = 252)	2 (1–3)
Designation	*n* (%)^b^
Physician	63 (16.0)
Nurse	261 (66.1)
Pharmacist	5 (1.3)
Dietician	3 (0.8)
Technician	23 (5.8)
Health educator	1 (0.3)
Physiotherapists	7 (1.8)
Therapist	21 (5.3)
Others	11 (2.8)
Hospital/center/department^*∗*^	
Comprehensive Cancer Center	16 (4.1)
National Neurosciences Institute	9 (2.3)
King Salman Heart Center	19 (4.8)
Obesity Endocrine and Metabolic Center	1 (0.3)
Women's Specialized Hospital	49 (12.4)
Children's Specialized Hospital	135 (34.2)
Rehabilitation Hospital	33 (8.4)
Main Hospital	101 (25.6)
Others	28 (7.1)
Level of education^*∗*^	
High school or diploma	69 (17.5)
Bachelor	272 (68.9)
Master or PhD	52 (13.2)
Marital status^*∗*^	
Single	143 (36.2)
Married	239 (60.5)
Divorced	11 (2.8)
Widow	1 (0.3)
Single marriage (monogamy)^*∗*^ (*n* = 252)	223 (88.5)
Number of children (parity, *n* = 252)^*∗*^	
0	41 (16.3)
1–3	166 (65.9)
>3	31 (12.3)
Number of abortions^*∗*^ (*n* = 252)	
0	157 (62.3)
1–2	63 (25.0)
>2	10 (4.0)
One or more stillbirths^*∗*^ (*n* = 252)	18 (7.1)
Any history of cervical cancer^*∗*^	9 (2.3)
First-degree relatives' history of cervical cancer^*∗*^	15 (3.8)
Second-degree relatives or friend with history of cervical^*∗*^	12 (3.0)

^a^Mean (standard deviation (SD))/median (interquartile range (IQR)). ^b^Frequency (percentage). ^*∗*^Data are missing in participants' age (*n* = 30), years of experience (24), age at marriage (163), number of pregnancies (150), hospital/center/department (4), level of education (2), marital status (1), single marriage (163), number of children (152), number of abortions (163), one or more stillbirths (164), history of cervical cancer (13), first-degree relatives' history of cervical cancer (19), and second-degree relatives or friend's history of cervical (31). In the calculation of percentages (%), the denominators include missing observations.

**Table 2:** Participants' knowledge about cervical cancer.

Items of the knowledge scale for cervical cancer	Frequency (%)^a^	95% CI^b^
Risk factors		
R1: multiple sexual partners^*∗*^	35 (8.9)	6.2–12.1
R2: early sexual intercourse^*∗*^	57 (14.4)	11.1–18.3
R3: HPV infection (human papillomavirus)^*∗*^	38 (9.6)	6.9–13.0
R4: infection with the human immunodeficiency virus (HIV)^*∗*^	54 (13.7)	10.4–17.5
R5: cigarette smoking^*∗*^	51 (12.9)	9.8–16.6
R6: ever used contraceptive methods^*∗*^	53 (13.4)	10.2–17.2
Vulnerability		
V1: women age >50 years^*∗*^	34 (8.6)	6.0–11.8
V2: reproductive age^*∗*^	55 (13.9)	10.7–17.7
V3: both of the above^*∗*^	62 (15.7)	12.3–19.7
Sign and symptoms		
S1: vaginal bleeding^*∗*^	27 (6.8)	4.6–9.8
S2: foul-smelling vaginal discharge^*∗*^	29 (7.3)	5.0–10.4
S3: contact bleeding^*∗*^	48 (12.2)	9.1–15.8
Prevention		
P1: avoiding multiple sexual partners^*∗*^	30 (7.6)	5.2–10.7
P2: avoiding early sexual intercourse^*∗*^	49 (12.4)	9.3–16.1
P3: screening and treatment^*∗*^	11 (2.8)	1.4–4.9
P4: avoid/quit cigarette smoking^*∗*^	39 (9.9)	7.1–13.2
P5: all of the above^*∗*^	39 (9.9)	7.1–13.2
What are the ways of screening		
WS1: Pap smear^*∗*^	17 (4.3)	2.5–6.8
WS2: visual inspection of cervix^*∗*^	25 (6.3)	4.1–9.2
WS3: human papillomavirus DNA testing^*∗*^	42 (10.6)	7.8–14.1
WS4: liquid-based cytology^*∗*^	75 (19.0)	15.2–23.2
WS4: there is no way of screening^*∗*^	31 (7.9)	5.4–11.0
Median (IQR) total score for the knowledge scale^Ϯ^	1 (0–4)	
Level of knowledge based on the total score		
Poor (score of 0–4)	311 (80.8)	76.5–84.5
Fair (score of 5–10)	58 (15.0)	11.6–18.8
Good (score of 11–20)	16 (4.2)	2.5–6.8

^a^Frequencies and percentage (%) for the “Yes” responses; percentages are computed with missing observations included in the denominator. ^b^95% confidence intervals in column 3 for the percentages (%) in column 2. ^Ϯ^Responses to each item in column 1 were recoded as Yes = 1 and No or Don't know = 0, and total score (0–20) was computed, and median total score (interquartile range (IQR)) is presented in the table. ^*∗*^Data are missing in R1 (for 22 participants), R2 (24), R3 (20), R4 (41), R5 (29), and R6 (51); V1 (39), V2 (50), and V3 (77); S1 (14), S2 (19), and S3 (24); P1 (23), P2 (29), P3 (19), P5 (29), and P6 (67); WS1 (20), WS2 (35), WS3 (67), WS4 (72), and WS5 (73).

**Table 3:** Participants' attitudes towards cervical cancer.

Statements describing attitudes of the participants towards cervical cancer	Agree, *n* (%)	Neither agree nor disagree, *n* (%)	Disagree, *n* (%)
A1: carcinoma of the cervix is highly prevalent and is a leading cause of deaths amongst all malignancies in Saudi Arabia^*∗*^	52 (13.2)	124 (31.4)	210 (53.2)

A2: any young woman including you can acquire cervical carcinoma^*∗*^	29 (7.3)	48 (12.2)	309 (78.2)

A3: carcinoma of the cervix cannot be transmitted from one person to another^*∗*^	79 (20.0)	39 (9.9)	265 (67.1)

A4: screening helps in prevention of carcinoma of the cervix^*∗*^	18 (4.6)	32 (8.1)	335 (84.8)

A5: screening causes no harm to the client^*∗*^	36 (9.1)	41 (10.4)	309 (78.2)

A6: screening for cervical cancer is not expensive^*∗*^	51 (12.9)	108 (27.3)	225 (57.0)

A7: if screening is free and causes no harm, will you screen?^*∗*^	15 (3.8)	29 (7.3)	342 (86.6)

*n* (%): frequencies (percentage) of participants; percentages are computed with missing observations included in the denominator. ^*∗*^Data are missing in A1 (for 9 participants), A2 (9), A3 (12), A3 (10), A5 (9), A6 (11), and A6 (9).

**Table 4:** Practice and knowledge of cervical cancer screening.

Statements for assessing knowledge and practice of cervical cancer screening	Frequency (%)^a^	95% CI^b^
KP1: yes—I have heard of Pap smear test for CCS^*∗*^	335 (84.8)	81.3–88.2
KP2: it is a useful tool for early detection of cervical cancer^*∗*^	343 (86.8)	83.1–90.0
KP3: age at which Pap smear test be started^*∗*^		
From birth	1 (0.3)	0.01–1.4
From puberty	23 (5.8)	3.7–8.6
From 20 years	74 (18.7)	15.0–22.9
From 30 years	173 (43.8)	38.8–48.8
After menopause	117 (29.6)	25.2–34.4
KP4: yes—I have undergone Pap smear test^*∗*^	103 (26.2)	21.9–30.9
KP5: if yes to above, interval for Pap smear test^*∗*^		
Monthly	6 (5.4)	2.0–11.4
Yearly	76 (68.5)	59.0–77.0
After menopause	3 (2.7)	0.5–7.7
Not sure	26 (23.4)	15.9–32.4
KP6: reasons if the test is not done^*∗*^		
I see no reason for the test	116 (48.5)	42.0–55.0
I am afraid of the procedure	41 (17.2)	12.6–22.5
I am afraid of the bad results	8 (3.4)	1.5–6.5
I do not know whom to consult for undergoing this test	27 (11.3)	7.6–16.0
Others (including multiple of the above)	47 (19.7)	14.8–25.2
KP7: best time for doing Pap smear test^*∗*^		
During menstrual flow	12 (3.0)	1.6–5.2
A week after period	249 (63.0)	58.1–67.8
During pregnancy	2 (0.5)	0.06–1.8
During breastfeeding	1 (0.3)	0.0–1.4
Not sure	119 (30.1)	26.0–34.9
KP8: Pap smear test should be done by^*∗*^		
Doctor	301 (76.2)	71.7–80.3
Trained nurse	29 (7.3)	5.0–10.3
Not sure	29 (7.3)	5.0–10.3
Others (including the 1^st^ two)	25 (6.3)	4.1–9.2
KP9: abnormality in Pap smear test, what should be done?^*∗*^		
Leave it to God and pray	2 (0.5)	0.0–1.8
Do some lab tests	312 (78.9)	74.6–82.9
Not sure	40 (10.1)	7.3–13.5
Others	31 (6.3)	4.19.2
KP10: benefits of Pap smear test^*∗*^		
Early detection of cervical cancer	175 (44.3)	39.3–49.4
Detection of any early abnormal changes in the cervix	132 (33.4)	28.8–38.3
Not sure	20 (5.1)	3.1–7.7
Above two	59 (14.9)	11.6–18.8
KP11: Pap smear test is painful^*∗*^	156 (39.5)	34.6–44.5
KP12: undergoing Pap smear test is a good practice^*∗*^	324 (82.0)	77.9–85.6
KP13: Pap smear test is done using^*∗*^		
Transvaginal ultrasound	40 (10.1)	7.3–13.5
Vaginal brushing	224 (56.7)	51.7–61.7
Not sure	89 (22.5)	18.5–27.0
Others	33 (8.4)	5.8–11.5
KP14: human papillomavirus vaccination is available in our institution^*∗*^	56 (14.2)	10.9–18.1
KP15: I have been vaccinated for human papillomavirus^*∗*^	22 (5.6)	3.5–8.3

^a^Frequencies and percentage (%) of participants' responses; percentages are computed with missing observations included in the denominator. ^b^95% confidence intervals in column 3 for the percentages (%) in column 2. ^*∗*^Data are missing in KP1 (for 2 participants), KP2 (4), KP3 (7), KP4 (2), KP5 (not applicable or missing: 284), KP6 (not applicable or missing: 156), KP6 (12), KP7 (11), KP9 (10), KP10 (9), KP11 (11), KP12 (9), KP13 (48), and KP14 (47).

**Table 5:** Relation of the total score for knowledge about cervical cancer (categorical^a^) with demographic variables.

Sociodemographic variables	Poor knowledge (score: 0–4)	Fair knowledge (score: 5–10)	Good knowledge (score: 11–20)	*p* values
	Median (IQR)	Median (IQR)	Median (IQR)	
Age (≤30 years vs. >30 years)	34 (30–41)	29 (25–37)	35 (28–37.5)	0.002
Age at marriage	26.5 (24–28)	27 (24–29)	26.5 (25.5–28)	0.701
Total experience in years (<10 years vs. ≥10 years)	11 (7–16)	8 (1–13)	10.5 (4–14.5)	0.004

	*n* (%)	*n* (%)	*n* (%)	

Level of education				
High school or diploma	56 (18.1)	8 (13.8)	4 (25.0)	0.485
Bachelor	216 (69.9)	40 (69.0)	9 (56.3)	
Master or PhD	37 (12.0)	10 (17.2)	3 (18.8)	
Designation/profession				
Nurses	218 (70.1)	31 (53.5)	8 (50.0)	0.030
Physicians	48 (15.4)	11 (19.0)	3 (18.8)	
Others	45 (14.5)	16 (27.6)	5 (31.3)	
Hospital/center/department				
Others	126 (40.9)	20 (34.5)	8 (53.3)	0.139
CSH	104 (33.8)	27 (46.6)	2 (13.3)	
Main Hospital	78 (25.3)	11 (19.0)	5 (33.3)	

^a^Knowledge of cervical cancer could not be determined for 10 participants due to missing data for some of the responses.

## Data Availability

The datasets used and/or analyzed during the current study are available from the KFMC research center through the corresponding author on reasonable request.

## References

[1] Bray F., Ferlay J., Soerjomataram I., Siegel R. L., Torre L. A., Jemal A. (2018). Global cancer statistics 2018: GLOBOCAN estimates of incidence and mortality worldwide for 36 cancers in 185 countries. *CA: A Cancer Journal for Clinicians*.

[2] Kingdom of Saudi Arabia Saudi Health Council Saudi Cancer Registry (2014). Cancer incidence report Saudi Arabia 2014. https://nhic.gov.sa/eServices/Documents/2014.pdf.

[3] ICO/IARC Information Centre on HPV and Cancer (2017). Saudi Arabia human papillomavirus and related cancers, fact sheet 2017. http://www.hpvcentre.net/statistics/reports/SAU_FS.pdf.

[4] Momenimovahed Z., Salehiniya H. (2017). Incidence, mortality and risk factors of cervical cancer in the world. *Biomedical Research and Therapy*.

[5] Braaten K. P., Laufer M. R. (2008). Human papillomavirus (HPV), HPV-related disease, and the HPV vaccine. *Reviews in Obstetrics & Gynecology*.

[6] Adegoke O., Kulasingam S., Virnig B. (2012). Cervical cancer trends in the United States: a 35-year population-based analysis. *Journal of Women’s Health*.

[7] Bray F., Loos A. H., McCarron P. (2005). Trends in cervical squamous cell carcinoma incidence in 13 European countries: changing risk and the effects of screening. *Cancer Epidemiology Biomarkers & Prevention*.

[8] Torre L. A., Bray F., Siegel R. L., Ferlay J., Lortet-Tieulent J., Jemal A. (2015). Global cancer statistics, 2012. *CA: A Cancer Journal for Clinicians*.

[9] Torre L. A., Islami F., Siegel R. L., Ward E. M., Jemal A. (2017). Global cancer in women: burden and trends. *Cancer Epidemiology Biomarkers & Prevention*.

[10] McClung N. M., Gargano J. W., Bennett N. M. (2019). Trends in human papillomavirus vaccine types 16 and 18 in cervical precancers, 2008–2014. *Cancer Epidemiol Biomarkers Prev*.

[11] World Health Organization (2013). *WHO Guidelines for Screening and Treatment of Precancerous Lesions for Cervical Cancer Prevention. WHO Guidelines Approved by the Guidelines Review Committee*.

[12] Jassim G., Obeid A., Al Nasheet H. A. (2018). Knowledge, attitudes, and practices regarding cervical cancer and screening among women visiting primary health care Centres in Bahrain. *BMC Public Health*.

[13] Hussain A. N., Alkhenizan A., McWalter P. (2016). Attitudes and perceptions towards HPV vaccination among young women in Saudi Arabia. *Journal of Family and Community Medicine*.

[14] Manji M. (2000). Cervical cancer screening program in Saudi Arabia: action is overdue. *Annals of Saudi Medicine*.

[15] Subramanian S., Trogdon J., Ekwueme D. U. (2010). Cost of cervical cancer treatment: implications for providing coverage to low-income women under the Medicaid expansion for cancer care. *Women’s Health Issues*.

[16] Ackerson K., Gretebeck K. (2007). Factors influencing cancer screening practices of underserved women. *Journal of the American Academy of Nurse Practitioners*.

[17] So V. H. T., Channon A. A., Ali M. M. (2018). Uptake of breast and cervical cancer screening in four Gulf Cooperation Council countries. *European Journal of Cancer Prevention*.

[18] Al Sairafi M., Mohamed F. A. (2009). Knowledge, attitudes, and practice related to cervical cancer screening among Kuwaiti women. *Medical Principles and Practice*.

[19] Mbamara S. U., Ikpeze O. C., Okonkwo J. E. (2011). Knowledge, attitude and practice of cervical cancer screening among women attending gynecology clinics in a tertiary level medical care center in southeastern Nigeria. *The Journal of Reproductive Medicine*.

[20] Mukama T., Ndejjo R., Musabyimana A., Halage A. A., Musoke D. (2017). Women’s knowledge and attitudes towards cervical cancer prevention: a cross sectional study in Eastern Uganda. *BMC Women’s Health*.

[21] Narayana G., Suchitra M., Sunanda G., Ramaiah J., Kumar B., Veerabhadrappa K. (2017). Knowledge, attitude, and practice toward cervical cancer among women attending obstetrics and gynecology department: a cross-sectional, hospital-based survey in South India. *Indian Journal of Cancer*.

[22] Aweke Y. H., Ayanto S. Y., Ersado T. L. (2017). Knowledge, attitude and practice for cervical cancer prevention and control among women of childbearing age in Hossana town, Hadiya zone, Southern Ethiopia: community-based cross-sectional study. *PLoS One*.

[23] Al Khudairi H., Abu-Zaid A., Alomar O. (2017). Public awareness and knowledge of pap smear as a screening test for cervical cancer among Saudi population in Riyadh city. *Cureus*.

[24] Al-Shaikh G. K., Almussaed E. M., Fayed A. A. (2014). Knowledge of Saudi female university students regarding cervical cancer and acceptance of the human papilloma virus vaccine. *Saudi Medical Journal*.

[25] Al-Shaikh G. K., Syed S. B., Fayed A. A. (2017). Effectiveness of health education programme: level of knowledge about prevention of cervical cancer among Saudi female healthcare students. *Journal of the Pakistan Medical Association*.

[26] Al-Darwish A. A., Al-Naim A. F., Al-Mulhim K. S. (2014). Knowledge about cervical cancer early warning signs and symptoms, risk factors and vaccination among students at a medical school in Al-Ahsa, Kingdom of Saudi Arabia. *Asian Pacific Journal of Cancer Prevention*.

[27] Jradi H., Bawazir A. (2019). Knowledge, attitudes, and practices among Saudi women regarding cervical cancer, human papillomavirus (HPV) and corresponding vaccine. *Vaccine*.

[28] Sait K. H. (2009). Attitudes, knowledge, and practices in relation to cervical cancer and its screening among women in Saudi Arabia. *Saudi Medical Journal*.

[29] CDC (2019). Morbidity and mortality weekly report. https://www.cdc.gov/mmwr/preview/mmwrhtml/mm6151a2.htm.

[30] (2018). Gardasil prescribing information. https://www.fda.gov/downloads/biologicsbloodvaccines/vaccines/approvedproducts/ucm111263.pdf.

[31] (2018). Cervarix prescribing information. https://www.fda.gov/downloads/biologicsbloodvaccines/vaccines/approvedproducts/ucm111263.pdf.

[32] Alsbeih G. (2014). HPV infection in cervical and other cancers in Saudi Arabia: implication for prevention and vaccination. *Frontiers in Oncology*.

